# Present and future of machine learning in breast surgery: systematic review

**DOI:** 10.1093/bjs/znac224

**Published:** 2022-08-10

**Authors:** Chien Lin Soh, Viraj Shah, Arian Arjomandi Rad, Robert Vardanyan, Alina Zubarevich, Saeed Torabi, Alexander Weymann, George Miller, Johann Malawana

**Affiliations:** School of Clinical Medicine, University of Cambridge, Cambridge, UK; Department of Medicine, Faculty of Medicine, Imperial College London, London, UK; Department of Medicine, Faculty of Medicine, Imperial College London, London, UK; Research Unit, The Healthcare Leadership Academy, London, UK; Department of Medicine, Faculty of Medicine, Imperial College London, London, UK; Department of Thoracic and Cardiovascular Surgery, West German Heart and Vascular Center Essen, University Hospital of Essen, University Duisburg-Essen, Essen, Germany; Department of Anesthesiology and Intensive Care Medicine, University Hospital of Cologne, Cologne, Germany; Department of Thoracic and Cardiovascular Surgery, West German Heart and Vascular Center Essen, University Hospital of Essen, University Duisburg-Essen, Essen, Germany; Research Unit, The Healthcare Leadership Academy, London, UK; Centre for Digital Health and Education Research (CoDHER), University of Central Lancashire Medical School, Preston, UK; Research Unit, The Healthcare Leadership Academy, London, UK; Centre for Digital Health and Education Research (CoDHER), University of Central Lancashire Medical School, Preston, UK

## Abstract

**Background:**

Machine learning is a set of models and methods that can automatically detect patterns in vast amounts of data, extract information, and use it to perform decision-making under uncertain conditions. The potential of machine learning is significant, and breast surgeons must strive to be informed with up-to-date knowledge and its applications.

**Methods:**

A systematic database search of Embase, MEDLINE, the Cochrane database, and Google Scholar, from inception to December 2021, was conducted of original articles that explored the use of machine learning and/or artificial intelligence in breast surgery in EMBASE, MEDLINE, Cochrane database and Google Scholar.

**Results:**

The search yielded 477 articles, of which 14 studies were included in this review, featuring 73 847 patients. Four main areas of machine learning application were identified: predictive modelling of surgical outcomes; breast imaging-based context; screening and triaging of patients with breast cancer; and as network utility for detection. There is evident value of machine learning in preoperative planning and in providing information for surgery both in a cancer and an aesthetic context. Machine learning outperformed traditional statistical modelling in all studies for predicting mortality, morbidity, and quality of life outcomes. Machine learning patterns and associations could support planning, anatomical visualization, and surgical navigation.

**Conclusion:**

Machine learning demonstrated promising applications for improving breast surgery outcomes and patient-centred care. Neveretheless, there remain important limitations and ethical concerns relating to implementing artificial intelligence into everyday surgical practices.

## Teaser

A systematic review evaluating the applications of machine learning in the field of breast surgery. We evaluate the current evidence and areas of application in order to improve patient outcomes. We further examine the limitations of the field and provide recommendations for future research in the field of machine learning and breast surgery.

## Background

Artificial intelligence (AI) refers to computer systems that mimic human cognitive functions and learn using large data sets^1^. Recent years have shown a dramatic development in these technologies in healthcare being employed in a wide variety of diagnostic and decision-making processes^2^. In an emerging era of big data, the scope and scale of patient data available and leaps in computational ability have allowed AI to develop and improve in its efficiency and applicability^3^.

AI technology is progressing rapidly, with support from healthcare professionals, industry, and governments^4^. Health care has adopted these technologies to improve patient outcomes, especially in the field of surgery. These technologies demonstrate unique potential in surgery with preoperative planning, patient outcome predictions, and even overcoming the challenges of the COVID-19 pandemic, as demonstrated by the recent COVIDSurg Collaborative addressing the impact of COVID-19 on patient mortality with a predictive model^5^.

AI encompasses many disciplines of computer learning, and a clinically relevant subtype of AI includes machine learning^1,6^. Machine learning focuses on using algorithmic packages and data to mimic the way humans learn^2^. The algorithms use data inputs to ‘learn’, uncovering associations in data sets via pattern recognition, repetition, and modification to make autonomous decisions and predict future outcomes. Common subsets of machine learning include prediction models, deep learning, and natural language processing^7,8^.

Breast surgery, a subspecialty within general surgery, is a field that has much to benefit from the advances in AI to provide the best patient care by surgical interventions in benign and malignant breast disease. Machine learning in breast surgery may involve these sets of models and methods to detect patterns in vast amounts of patient data, extract appropriate information, and use it to perform decision-making under uncertain conditions^9^. The potential applications of machine learning are significant, and breast surgeons must strive to be informed with up-to-date knowledge and applications of this subset of AI within their speciality^10,11^.

The aim of this review was to study the applications of machine learning in breast surgery. Past reviews in other surgical specialities have been written, but none specifically for breast surgery. This review is designed to evaluate closely the current applications by synthesizing current research, and to catalyse future research efforts in this advancing field.

## Methods

### Literature search strategy

**Fig. 1:**
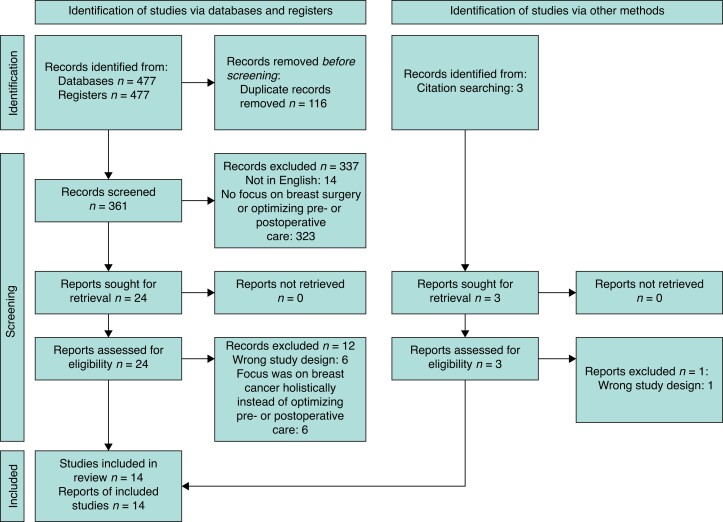
PRISMA flowchart

This systematic review was conducted in accordance with the Cochrane Collaboration and PRISMA guidelines^12^. A literature search was conducted including Embase, MEDLINE, Cochrane, PubMed, and Google Scholar from inception to December 2021 (*Fig. 1).* The search terms used were (Machine Learning OR Artificial Intelligence OR Deep learning OR Decision Trees OR Neural Networks) AND (Breast Surgery OR Mastectomy OR Breast-conservative Surgery OR Breast reduction OR Breast reconstruction OR Breast augmentation OR Breast Cancer Surgery). Further articles were identified through use of the ‘related articles’ function on MEDLINE and a manual search of the references lists of articles found in the original search. The only limits used were the English language and the aforementioned time frame.

### Study inclusion and exclusion criteria

All original articles reporting the use of machine learning in breast surgery were included. Studies were considered if they presented machine learning models with the aim of supporting breast surgery or providing a prognosis for an intervention, either used by itself or with other methods. There were no geographical restrictions. Studies were excluded from the review if the quality of available data and data inconsistencies precluded valid extraction, or if the study was performed in an animal model. Case reports, reviews, abstracts from meetings, and preclinical studies were excluded. Machine learning is a highly erratic and dynamic field. This review contains literature published over a 5-year time period between 2017 and 2021 inclusive, with significant technology changes even in the 5 years preceding conduction of this review. As a result, there have been many advancements that have superseded some of the points raised in earlier literature, and care was taken to recognize each study in the unique context of its publication year. It was ensured that any outdated findings did not shape the review. By following the aforementioned criteria, two reviewers (C.L.S. and V.S.) independently identified articles for further assessment following title and abstract review. Disagreements between the two reviewers were resolved by a third independent reviewer (A.A.R.). Potentially eligible studies were then retrieved for full-text assessment.

### Data extraction and critical appraisal of the evidence

The full texts of retrieved articles were read and reviewed by two authors (C.L.S. and V.S.), and the inclusion or exclusion of studies was decided unanimously. When there was disagreement, a third reviewer (A.A.R.) made the final decision. Using a pre-established protocol, the following data were extracted: first author; study type and characteristics; number of patients; population demographics; type of procedure; category of machine learning method used; method of machine learning implemented; and main reported outcomes.

### Risk of bias

**Fig. 2:**
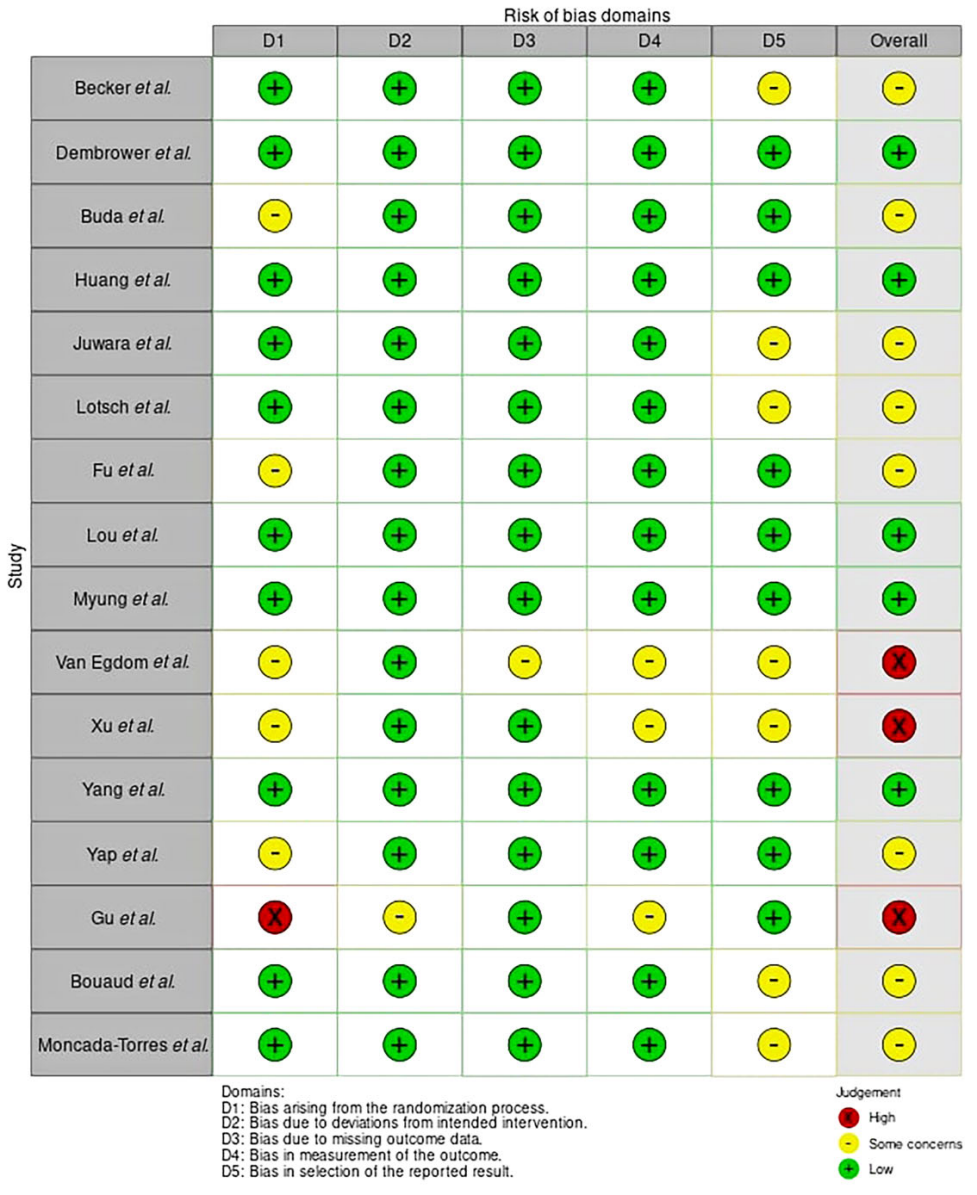
Risk of bias diagram

The risk of bias of the selected articles was evaluated by two independent reviewers (C.L.S. and V.S.) using an adapted Cochrane Collaboration Risk of Bias tool *(Fig. 2)*. The methodological quality of the studies were assessed based on the following domains: participation; response; outcome measurement; statistical analysis and reporting; and confounding. An overall grading of low, medium, or high risk of bias was then applied. Additionally, the limitations of this systematic review are more expansively outlined in *Appendix S1*.

## Results

### Study selection

The literature search identified 477 articles; following the removal of duplicates, 361 were screened. The full texts of 24 articles were reviewed and assessed in accordance with the inclusion and exclusion criteria. Following critical appraisal, a total of 14 studies were included in this review, featuring 73 847 patients.^12–25^  *Figure 1* illustrates the entire study selection process. A summary of the studies collected and their respective designs, type of machine learning mode used, and its implementation, as well as the main reported outcomes are presented in *Table 1*.

**Table 1:** Studies included assessing machine learning in breast surgery

Study	Study design	Type of procedure	Category of machine learning used	*n*	Method of machine learning implemented	Main reported outcomes
**Becker *et al*.^13^**	NM, NR, NP	Mammography	Image analysis	3228	Patients experiencing mammography at the institution were chosen to form the dataset, which was used to train the neural network An external data set from the Breast Cancer Digital Repository was used for testing, which was appraised by three radiologists	One radiologist showed nearly equivalent performance to the network (0.83, *P* = 0.170) and the other two performed significantly better (0.91 and 0.94 respectively, *P* < 0.016) The neural network's performance of 0.82 did not differ significantly between radiologist performance. The neural network behaved less specifically and more sensitively than humans throughout
**Dembrower *et al.*^14^**	M, NR, NP	Mammography	Screening	7364	A cohort of mammograms were selected in a way that mimicked frequency in reality. The AI cancer detector algorithm used had been pretrained The software generates underlying image-level prediction scores for tumour presence. From this, cut-off points were established to insert patients into two novel work streams: on missed and additionally detected cancer	For 60%, 70%, or 80% of women with the lowest AI scores in the negative radiologist stream, 0.3% (95% c.i. 0.0–4.3)/2.6% (95% c.i. 1.1–5.4) of screen-detected cancers would be missed, respectively. For the 1% or 5% of women possessing highest AI scores in the ‘enhanced assessment’ stream, 12% or 27% of subsequent interval cancers, respectively, and 14% or 35% of next-round screen-detected cancers, respectively, may have also been able to be detected additionally
**Buda *et al*.^15^**	M, NR, NP	Digital breast tomosynthesis	Deep learning algorithm	16 802	16 802 digital breast tomosynthesis examinations that had one reconstruction view between 26 August 2014 and 29 January 2018 were analysed These were subdivided into four groups and further split into training and testing sets for the evaluation of the deep learning model	The deep learning model trained reached a breast-based sensitivity at 65% (39 of 60; 95% c.i. 56–74) on the test set. This was at 2 false positives per DBT volume
**Huang *et al.*^16^**	M, NR, NP	Breast cancer surgery	Predictive model	3632	This study compared three models (MLR, Cox, ANN) for 3632 postoperative patients with breast cancer between 1996 and 2010 An estimation data set trained the model, and a validation data set helped to evaluate the performance of the model. Sensitivity analysis allowed the comparison of input variables for the model's predictions	Overall, the ANN model performed best over the MLR and Cox models for predicting 5-year breast cancer mortality postoperatively Age, Charlson Comorbidity Index (CCI), chemotherapy, radiotherapy, hormone therapy, breast cancer surgery volumes of hospital and surgeon were significantly associated with 5-year breast cancer surgery; the latter was the most significant
**Juwara *et al.*^17^**	NM, NR, P	Breast cancer surgery	Predictive model	195	Six machine learning algorithms (least square, ridge, elastic net, random forest, gradient boosting, and neural net) aided primary analysis of the identification of predictors of DN4 interview score (index for neuropathic pain) Models were compared. A logistic classification model was created for neuropathic pain using the predictor outcomes of the primary analysis	Anxiety, type of surgery, preoperative baseline pain, and acute pain on movement predicted DN4 score most pertinently. Anxiety had the most significant association with neuropathic pain The least square regression model compared well with the random forest model and neural network model. The gradient boosting model performed better than all the other models
**Lötsch *et al.*^18^**	NM, NR, P	Breast cancer surgery	Supervised machine learning prediction	1000	Machine learning helped establish a short questionnaire to identify pain to the same predictive level as pre-existing full-form questionnaires The predictors were trained via the full set of items in Beck's Depression Inventory (BDI), Spielberger's State-Trait Anxiety Inventory (STAI), and the State-Trait Anger Expression Inventory (STAXI-2) Machine learning extracted features of these to create predictors with a lower item number	A seven-item set produced via Machine learning that comprised 10% of the original questions from the STAI and BDI performed the same as the full questionnaires in predicting development of persistent postoperative pain
**Fu *et al.*^19^**	M, NR, NP	Breast cancer surgery	Detection	355	A mobile health system allowed data to be collected based on real-time symptom reporting Both statistical and Machine learning processes were employed for data analysis. Regarding the latter, five classical algorithms were compared: Decision Tree of C4.5, Decision Tree of C5.0, gradient boosting model (GBM), ANN, and SVM	Using Machine learning to compare different algorithms is a viable concept. The ANN performed best for detecting postoperative development of lymphedema (accuracy 93.75%, sensitivity 95.65%, specificity 91.03%)
**Lou *et al.*^20^**	M, NR, NP	Breast-conserving surgery, mastectomy with reconstruction	Predictive model	1140	The cases in this study were divided into a training data set to develop the machine learning model, a testing data set for internal validation, and an externally validating data set After training, outputs of the model were taken for each training set. Accuracy in predicting breast cancer recurrence within 10 years was compared	The ANN model performed significantly best of all models based on sensitivity, specificity, PPV, NPV, accuracy, and AUROC values Surgeon volume followed by hospital volume and tumour grade were, in that order, the best predictors of recurrence of breast cancer within 10 years
**Myung *et al.*^21^**	NM, NR, NP	Microsurgical reconstruction: muscle-sparing type TRAM and DIEP abdominal flaps	Predictive model	568	Neuralnet and RSNNS machine learning packages were applied to compare prediction accuracy, sensitivity, specificity, and predictive power (AUC) for predicting factors that raise abdominal flap donor site complications (against logistic regression). 13 variables suggested to influence donor site complication rates were evaluated	Neuralnet performed most optimally of all the packages Fascial defect, history of diabetes, muscle-sparing type, and presence or absence of adjuvant chemotherapy all significantly affected complication rate of donor sites Upon statistical analysis, high-risk group complication rates were significant compared with the low-risk group
**van Egdom *et al.*^22^**	NM, NR, NP	Breast cancer surgery	Predictive model	764	Various patient data variables were available Machine learning methods (GLM regression, SVM, single-layer ANNs, and deep learning) evaluated preoperative prognostic factors of age, medical status, tumour characteristics, and (neo)adjuvant treatment indications or treatment characteristics	No relationship was determined between predictors and outcomes, rendering the model akin to the outcomes’ respective population prevalence. Combining variables and simultaneously reducing dimensions did not yield significant changes
**Yang *et al.*^23^**	NM, NR, NP	Breast cancer surgery	Predictive model	1061	This study posed a predictive model of breast cancer recurrence based on clinical, nominal, and numeric features Six features from an initial data set were identified for further processing and resampling AdaBoost and cost-sensitive learning packages predicted the risk of recurrence and evaluated performance	AdaBoost reaches an accuracy of 0.973 and sensitivity of 0.675. A combination of AdaBoost and cost-sensitive learning poses a model with a reasonable accuracy of 0.468 and very high sensitivity of 0.947. Hence, the model is can be used to support early intervention
**Yap *et al.*^24^**	M, NR, NP	Breast ultrasound detection	Image analysis	469	The study employs a deep learning method for breast ultrasound ROI detection and lesion localization. Transfer learning is used to owing to unavailability of datasets and a novel three-channel artificial RGB method is applied for performance improvement This proposed method is evaluated and compared using an individual and composite data set	Faster RCNN outperformed a computer vision object detection algorithm indicating viability for use in BUS lesion localization IoU (equivalent to Dice Coefficient Index) should be used in lesion detection owing to its reliability
**Moncada-Torres *et al.*^25^**	M, NR, P	Breast-conserving surgery, mastectomy	Predictive model	36 658	Data of patients who underwent curative breast surgery was used to compare the performance of CPH analysis with machine learning modes (random survival forests, survival support vector machines, and XGB) for survival predictions	Machine learning models perform to at least the same standard as classical CPH regression and even better for some models (XGB). Furthermore, SHAP values were used successfully as a form of explainable machine learning to provide detail on how the models’ predictions are made
**Sidey-Gibbons *et al.*^26^**	NM, NR, P	Breast cancer surgery	Predictive model	611	A set of machine learning algorithms (neural network, regularized linear model, SVM, and a classification tree) were trained and tested to make predictions of financial toxicity in a data set The data were split into samples for the training and testing sets, prior to assessment of predictive performance	Machine learning packages accurately predicted financial toxicity in this context demonstrating an AUROC of 0.85, accuracy of 0.82, sensitivity of 0.85, and specificity of 0.81 Neoadjuvant therapy and autologous reconstruction were ascertained as key indicators of financial toxicity Radiation and tumour grade showed no effect

NM, non-multicentre; NR, non-randomized; NP, non-prospective; AI, artificial intelligence; M, multicentre; MLR, multiple logistic regression; ANN, artificial neural network; P, prospective; BDI, Beck Depression Inventory; STAI, Spielberger’s State–Trait Anxiety Inventory; STAXI-2, State–Trait Anger Expression Inventory; GBM, gradient boosting model; SVM, support vector machine; PPV, positive predictive value; NPV, negative predictive value; AUROC, area under the receiver operator curve; TRAM, transverse rectus abdominis muscle; DIEP, deep inferior epigastric artery perforator; AUC, area under the curve; GLM, general linear model; ROI, region of interest; BUS, breast ultrasound; IoU, Intersection over Union; XGB, extreme gradient boosting; CPH, Cox proportional hazards; SHAP, Shapley Additive Explanation.

Nine studies^16–18,20–23,25,26^ described examples of machine-learning based predictive modelling, comprising 45 792 patients and included a conglomerate of different modelling methods. Predictive modelling was the most common use in terms of recorded studies and patient volume. The use of machine learning in imaging was also described. Three studies^13,15,24^ comprising 20 499 patients described examples of machine learning within an image-based context for analysis and detection. Different machine learning models were applied in all the studies. One study^14^ including 7364 patients described a case of machine learning within screening and triaging. Furthermore, there was one study^19^ of 355 patients that described a machine learning network utility for detection purposes.

### Challenges and recommendations

**Fig. 3:**
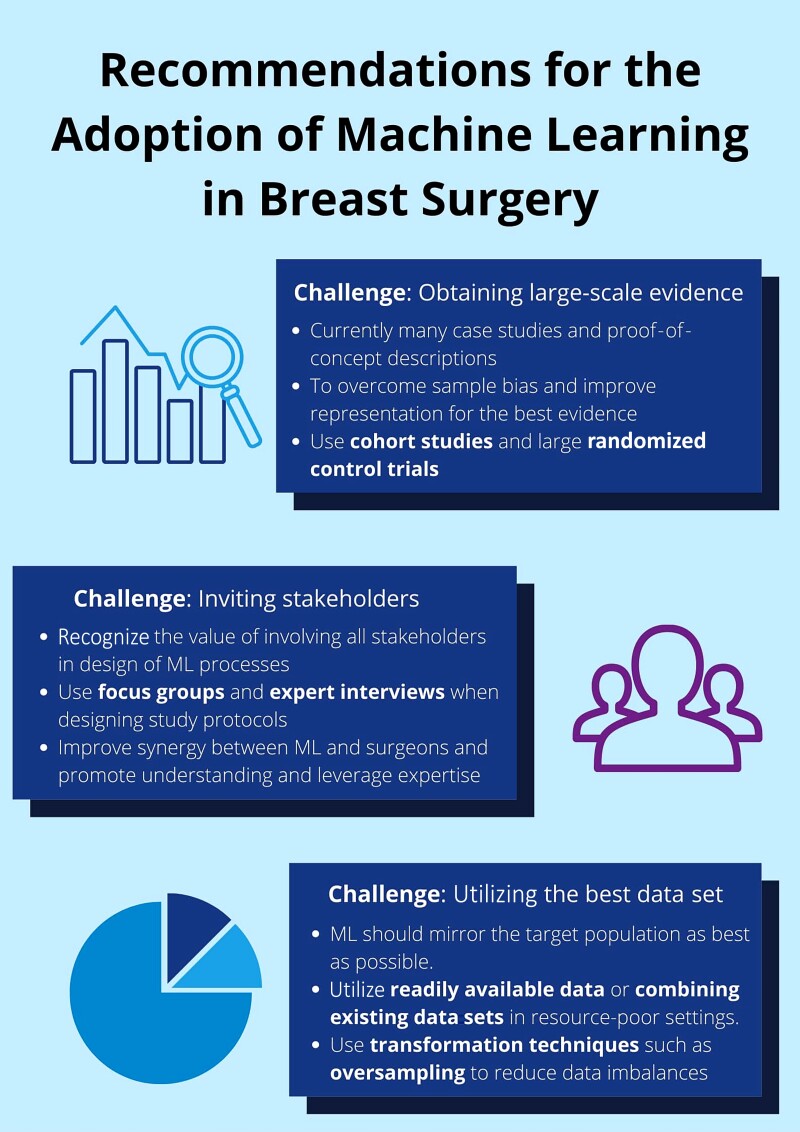
Challenges and recommendations in machine learning research within breast surgery

*Figure 3* summarizes the main challenges and respective recommendations developed from the literature with regard to future research in the field of machine learning and its application in breast surgery. The recommendations should be taken into the context of future research studies and be considered with all the information available.

## Discussion

This systematic review provides a summary of machine learning and AI within breast surgery. Although the results demonstrate successes with different approaches, these must be considered in the context of their limitations as most applications remain at the ‘proof-of-concept’ stage.

There is evident value to the use of machine learning in preoperative planning in both cancer and an aesthetic context. The diagnosis and detection of pathology is fundamental in preoperative planning for breast cancer surgery. The use of image analysis in clinical applications such as breast imaging, digital pathology, and surgical planning is well described in the literature. Moreover, the use of modern imaging techniques in screening and diagnostics has led to the development of many machine learning solutions. A retrospective study from Becker *et al*.^13^ on mammography diagnostics using a neural network image analysis software demonstrated an equivalent performance of the neural network when compared to radiologists. The results differed between radiologists, but the neural network showed an increased sensitivity of 72 per cent *versus* a 66.7 per cent average across radiologists overall. Corroborating these findings, a retrospective simulation study from Dembrower *et al*.^14^ and diagnostic study from Buda *et al*.^15^ demonstrated similar levels of prediction using different means of deep learning models such as a commercial AI-based cancer-detector algorithm. Cancer detection via data sets allows specific interventions that increase the efficiency of surgical planning and treatment decisions. The employment of specific machine learning technologies, including the Faster-RCNN with Inception-ResNet-v2 deep-learning framework, described by Yap *et al*.^24^, for ultrasound breast images, allows surgeons to focus on the relevant area of the breast.

The models that exist promise a reduction in the numbers of biopsies and bring efficiency to radiology interpretations while reducing workload. Reducing overdiagnosis, morbidity, and increasing time efficiency of breast imaging is a future aspiration. Diagnostic and prognostic applications in imaging and pathology have been studied greatly, with a wide evidence base of applied research. The transfer of imaging information to the operating theatre in order to more accurately localize cancer during surgery can further aid the field.

However, most applications of machine learning within breast surgery centre around the prediction of patient outcomes. This is coherent with the wider applications of machine learning within modern surgery^28–30^. In multiple instances machine learning was employed alongside traditional statistical modelling for prediction. Indicative of the success of machine learning, the former outperformed the latter in every highlighted example, and thus replicates the successes seen across other surgical subspecialties, mostly prominently neurosurgery^31^. This is particularly evidenced via the longitudinal study by Huang *et al.*^16^, which demonstrated accurate assessment of 5-year mortality after breast cancer surgery using machine learning algorithms. Although machine learning packages have been substantiated to show marked improvements to pre-existing models, including, but not limited to, least-square regression and Cox regression^17,25^, these still remain limited and relatively novel.

The studies included in this review demonstrated high heterogeneity in the form of machine learning applied. Some models have been more consistent and accurate than others. Artificial neural networks, algorithms that have been modelled after the human brain and nervous system^32^, were dependable across the scope of this review. This is exemplified most prominently in the study by Lou *et al*.^20^, where the artificial neural network package demonstrated the highest prediction performance index. This provides support to previous literature describing the effectiveness of artificial neural networks in other clinical contexts^32–34^. Artificial neural networks are better adapted to deal with problematic inputs, specifically in cases where this may be noisy or incomplete. As an example, a 93.75 per cent accuracy rate of identifying postoperative lymphedema in patients with breast cancer was shown by Fu *et al*.^19^. Many medical databases, with the scale where a machine learning model can be realistically derived, contain non-normally distributed data. This is challenging to many forms of modelling that assume normal distribution within a dataset^23^. As artificial neural networks are applicable to well-correlated data that are not necessarily natively normally distributed, they are more transferrable and provide greater potential for use in wider treatment contexts beyond breast cancer surgery.

The capacity for machine learning in breast surgery can extend past predicting outcomes and pivot towards providing more holistic patient assessment such as the prediction of postoperative pain^17,18^. Machine learning creates opportunities for more efficient pain assessment that can be undertaken immediately postoperatively, in comparison to pre-existing tools that require time-heavy questionnaires and extensive clinician–patient interaction. This serves great utility in the context of a healthcare system, where both time and human resources are often limiting factors. As neuropathic pain can be debilitating for patients, early prediction can allow clinicians to better optimize postoperative care.

A factor repeatedly indicated by machine learning models is surgeon volume as the largest predictor of reduced breast cancer recurrence after surgery^16,20^. Decision analysis and modes of machine learning within this context^25,36^ would allow this to further improve decision-making for surgeons with lower operation volumes. In line with this, the evidence suggests that some machine learning packages can outperform even the most experienced surgeon, and therefore may provide a template for replication by surgeons of all grades.

Additional applications of machine learning in breast surgery can be considered, although most of these remain conceptual. Decision-making in modern medicine is complex owing to the increasing availability of data to consider before treatment^37^. Advances in medical knowledge, including that of well-researched novel therapies and surgery, dramatically increase the potential treatment choice algorithms. Decision support systems are well described, including the DESIREE project^38^, which provides physicians with decision support modules. Other examples are decision-support models regarding recurrence prediction and support systems that encompass AI and information visualization^39^.

Computer vision for object and scene recognition could support surgical techniques, with patterns and associations used in planning, anatomical visualization, and surgical navigation. The exploration of machine learning systems that perform or directly complement surgery is rapidly developing, and may available in the imminent future. Real-time decision-making supported by machine learning provides exciting opportunities^40^.

Despite the benefits and potential applications in the field, clinicians must consider the potential limitations and risks of the technology. It is important to avoid overt optimism, and instead focus realistically on the barriers to implementation of machine learning clinically^41^. Machine learning and AI are limited by the lack of accurate and unbiased data collection and input. If data-input bias is evident, predictions may easily become unreliable. Examples include systematic biases due to non-representative predictions for patient groups not represented in research^42^. This review provides evidence in support of theoretical machine learning applications; however, as outlined by Manlhiot *et al*.^43^, care must be taken to recognize that these might not be clinically representative. The described machine learning models rely on heavily curated datasets with relatively few implementation obstacles, which is in vast contrast to data sets available in clinical practice. Moreover, machine learning can exhibit ‘black box’ characteristics, with incomprehensibly complex algorithms for their outputs. The learning mechanisms of some machines have been difficult to reproduce, and it has been difficult to justify certain decisions. Measures taken in the programming and comparison with clinical gold standards can circumvent this challenge. The challenges surrounding the complexity of machine learning in its current state renders it unimplementable without expertise and specialist knowledge. Explainable machine learning, whereby the system is able to justify how it made its predictions on a level that is comprehensible to a clinician^44,45^, might be a potential solution.

In addition, considerations of collaboration with other stakeholders in the implementation of the technology, in order to ensure data are interpreted correctly and applied in the correct manner, are of paramount importance. Planning the most safe and beneficial method of implementation, with close collaboration of healthcare professionals and machine learning and AI experts in a multidisciplinary approach, is required to ensure the best outcomes for all. In addition, the engagement of patients with breast cancer in decisions where patients can be informed are important.

Economic considerations, job losses, and the lack of human element pose additional ethical dilemmas. Machine learning may be stifled from practical implementation in breast surgery due to infrastructural shortcomings (with regard to both hardware and software) in the postdeployment management, a phenomenon that has been described within cardiology^43^. Ethicolegal and social issues, including the lack of regulatory structures surrounding machine learning technology, must be addressed and solutions explored. Financial considerations and the accessibility of this technology in low- and middle-income countries should also be considered.

The most favourable studies included in this review included high sample sizes and were multicentric. Many studies circumnavigated the challenge of a low centre sample size by combining with registry data to build their respective algorithms. Potential prospective solutions may also have basis in the concept of federated learning, an machine learning approach that allows an algorithm to combine data collectively from multiple centres without physical exchange of the data^46^. Hence, it is clear that any future approaches should ensure that this collaborative approach is undertaken as standard. Many studies encountered additional issues with data imbalances. To correct this, as an example, Myung *et al*. applied the ROSEs and SMOTE oversampling technique^47^. Future studies may consider employing this to increase the validity and generalizability, and consequently the probability of success^21^.

Machine learning must be recognized as still being in a trial phase: it is not perfect and is subject to multiple flaws^26^. The current literature provides fundamental foundations to its applicability, but future approaches must consider clinical relevance at their core in order to facilitate greater data-based shared patient–clinician decision-making in breast surgery. Hence, there is sufficient groundwork to construct prospective randomized studies to observe the impact of machine learning in clinical practice.

## Supplementary Material

znac224_Supplementary_Data

## Data Availability

Data collection form and search results are available on enquiry to the corresponding author (A.A.R).
