# Supplementary material for: Present and future of machine learning in breast surgery: systematic review
Source: Br J Surg. 2022 Aug 10;109(11):1053–62. doi: 10.1093/bjs/znac224 (PMC10364755; doi:10.1093/bjs/znac224)
Supplement: znac224_Supplementary_Data [file znac224_supplementary_data.zip › Supplementary_Appendix_1.docx]

**Appendix S1: Limitations of the Review & Field of Study**

This systematic review is additionally subject to its own intrinsic limitations. Given the format of the studies included in this review, a blinding methodology was unviable and this may create a source of potential cognitive bias across some of the included studies.

This review was further limited by a significantly unbalanced set of sample sizes, with a range between 195 and 36,658 patients across the studies included within it. Six of the studies contain sample sizes with 1000 or fewer patients. Machine learning algorithms are notoriously data-driven and perform optimally in scenarios where training models are developed using larger databases, moreover that is an assumed condition for the algorithm’s development (1,2). A common danger with smaller databases is that they contain a disproportionately larger qauntity of poor data points including noise, as aforementioned, as well as outliers and random errors. As a result, they encourage the principle of overfitting whereby a machine learning algorithm models the data to include these erroneous points and incidentally describes random errors rather than the interplay between variables in a dataset (3). This forces the outcomes of these algorithms to be far less generalisable. This review invariably considered all studies uniformly, yet some studies held greater merit on account of their generalisability and would have been more useful comparators over those with lower sample sizes, which was not accounted for in this review. The van Egdom et al. pilot study included in this review itself describes a case where the development of a machine learning algorithm failed and cites its aim to reduce the number of data dimensions as an inherent limitation (4). Nonetheless, as artificial neural networks aim to account for overfitting and this was the most common machine learning technique employed across all studies, this should not significantly query the overarching findings of the review.

The term ‘publication bias’ references a common occurrence in systematic reviews where published academic literature is far likelier to report statistically significant findings over insignificant findings (5). This review inevitably finds itself prone to this phenomenon. Furthermore, the incorporation of randomised controlled trials into this review may have improved its validity, but this was restricted by the availability of data. Selection bias may also form a component of the studies included in this review.

**References**

1. van der Ploeg T, Austin PC, Steyerberg EW. Modern modelling techniques are data hungry: a simulation study for predicting dichotomous endpoints. BMC medical research methodology [Internet]. 2014 [cited 2021 Dec 7];14(1). Available from: <https://pubmed.ncbi.nlm.nih.gov/25532820/>
2. Al-Jarrah OY, Yoo PD, Muhaidat S, Karagiannidis GK, Taha K. Efficient Machine Learning for Big Data: A Review. Big Data Research. 2015 Sep 1;2(3):87–93.
3. Ying X. An Overview of Overfitting and its Solutions. Journal of Physics: Conference Series [Internet]. 2019 Feb 1 [cited 2021 Dec 7];1168(2):022022. Available from: <https://iopscience.iop.org/article/10.1088/1742-6596/1168/2/022022>
4. van Egdom LSE, Pusic A, Verhoef C, Hazelzet JA, Koppert LB. Machine learning with PROs in breast cancer surgery; caution: Collecting PROs at baseline is crucial. The Breast Journal [Internet]. 2020 Jun 1 [cited 2021 Dec 7];26(6):1213–5. Available from: <https://onlinelibrary.wiley.com/doi/full/10.1111/tbj.13804>
5. Song F, Hooper L, Loke YK. Publication bias: what is it? How do we measure it? How do we avoid it? Open Access Journal of Clinical Trials [Internet]. 2013 Jul 4 [cited 2021 Dec 7];5(1):71–81. Available from: <https://www.dovepress.com/publication-bias-what-is-it-how-do-we-measure-it-how-do-we-avoid-it-peer-reviewed-fulltext-article-OAJCT>
